# Functional Characterization of* 9-/13-LOXs* in Rice and Silencing Their Expressions to Improve Grain Qualities

**DOI:** 10.1155/2016/4275904

**Published:** 2016-06-15

**Authors:** Moytri RoyChowdhury, Xiaobai Li, Hangying Qi, Wenxu Li, Jian Sun, Cheng Huang, Dianxing Wu

**Affiliations:** ^1^Department of Horticulture, Washington State University, Pullman, WA 99164, USA; ^2^State Key Lab of Rice Biology, International Atomic Energy Agency Collaborating Center, Zhejiang University, Hangzhou, Zhejiang 310029, China; ^3^Zhejiang Academy of Agricultural Sciences, Hangzhou, Zhejiang 310021, China; ^4^Agricultural Extension Extending Stations, Shaoxing & Zhuji Agricultural Bureau, Shaoxing, Zhejiang 312000, China; ^5^Institute for Wheat Research, Henan Academy of Agricultural Sciences, Zhengzhou, Henan 450002, China

## Abstract

Lipoxygenases (LOXs) are involved in oxidative rancidity and render rice unsuitable for human consumption. Here, RNA interference- (RNAi-) induced gene expression inhibition was used to analyze the functions of the bran/seed-specific LOXs in rice.* r9-LOX1* and* L-2 *(9-LOX category) were the candidate genes expressing a bran/seed-specific LOX, while* RCI-1* was (13-LOX category) a plastid-specific LOX. Real-time PCR showed that three LOXs were cultivar/tissue specific expression on a certain level.* r9-LOX1* and* L-2* were generally much higher in active bran/seed than in stabilized bran, mature seed, and regenerated plant.* RCI-1 *was barely expressed in seed. In transgenic lines,* r9-LOX1*, as well as* L-2*, expression was dramatically downregulated, compared to the nontransgenic controls. SPME/GC-MS analysis of* r9-LOX1* RNAi transgenic lines showed 74.33% decrease in nonanal content (formed during oxidation of linoleic acid by lipoxygenase), but 388.24% increase in acetic acid and 184.84% hexanal (direct products of 13-LOX). These results indicate that* r9-LOX1* positively regulates the amount of nonanal but negatively regulates acetic acid and hexanal. The negative regulation may be due to a mechanism of negative feedback between LOX family members. The information will help comprehensively understand the function of the bran/seed-specific LOXs,* r9-LOX1*, and improve the storage quality in the future.

## 1. Introduction

Lipoxygenases (LOXs) ubiquitously exist in the seeds of many plant species. Seed LOXs may be involved in the fatty acid peroxidation, lipids storage, production of growth regulators, responses to pathogens, and nitrogen storage [1]. The influence of LOXs on favor quality is reported in soybean and rice. Soybean LOXs are involved in the production of volatile compounds (such as n-hexanal) which are associated with grassy beany and rancid off-flavors in soybean and soy foods [2]. In stored rice seeds, LOX activities are also present [3], and hexanal is the predominant component of stale off-flavors derived from lipid peroxidation [4]. Rice seed* LOXs* are also involved in lipid peroxidation in rice seeds [5]. Since the absence of* LOXs* can alleviate seed lipid peroxidation,* LOXs* are always the targets of genetic modifying during molecular breeding. Soybean “IA2025,” a* LOX* triple null-mutation soybean line, can be used to produce tofu and soymilk with improved flavor and sensory qualities [6–8]. In barley, a* LOX-1* deficient line “*DaikeiLM1*” is gained from “*Karl M2*” through sodium azide treatment [9]. This mutated line can effectively improve flavor stability in beer [10]. In rice, the absence of sLOX3 in the seeds of “*DawDam*” results in less stale flavor [11].

According to the differences of substrates at carbon 9 or 13 of the hydrocarbon backbone, LOXs are classified into 9-LOXs and 13-LOXs [12]. Some plants may have predominant 9-LOX or 13-LOX activity, while some other plants may have equally 9-LOX and 13-LOX activities. According to the protein sequences, LOXs are classified into three types as described by Mizuno et al. [13]: Type I is localized in chloroplast and stress inducible; Type II is localized in cytoplasm, derived from dicots, and not stress inducible; Type III is localized in the cytoplasm, derived from monocots and related to seed germination. Type I LOXs have a transit peptide, lacking in Type II and Type III LOXs. Three isozymes in Type III LOXs (LOX1/L-1, LOX2/L-2, and LOX3/L-3) have been identified in* Oryza sativa* embryos. Among them, LOX3 is the most abundant one [14]. The catalytic LOX domains in both Type II and Type III LOXs have oxygen binding and oxylipin synthesis sites. The enzyme r9-LOX1 belongs to Type III and is the focus of this study. Three Type III LOXs (*L-1*,* L-2*, and* L-3*) in developing rice seeds have been identified [15].* L-2* (Type III) derives from 3-day-old seedlings [16], while* RLL* (Type I) is isolated from rice leaves [17].* L-3* (Type III) is the major component of these isozymes and accounts for 80–90% of their total activities [18]. The lack of* L-3* in* DawDam* leads to a decrease of lipid peroxidation and the alleviation of stale flavor accumulation by hexanal, as well as pentanal and pentanol compounds during storage [19]. Unfortunately,* DawDam* could not be used for rice production due to its poor agronomic characters [18]. It is speculated that the lack of LOX-3 may affect the metabolism in the whole plant.

In order to effectively improve rice grain storage qualities, inactivation of some lipase and/or LOXs should be achieved without compromising nutritional value and agronomic qualities. RNAi could be an ideal method to achieve this goal based on its applications on improving the nutritional quality of various crops [20]. Therefore, the objectives of this study were to select two candidate seed/bran* LOXs* gene(s), develop suitable constructs for stable RNAi in rice, and study the potential benefits of silencing bran/seed-specific LOXs.

## 2. Materials and Methods

### 2.1. Plant Materials

The japonica rice cultivar “LaGrue” was used to clone target genes and develop transgenic plants. The tissues from DawDam, Taipei-309, and transgenic lines were used for gene expression analysis.

### 2.2. Genomic DNA Extraction and PCR Analysis

Genomic DNA was extracted from the leaf tissues of* Taipei*-*309* seedlings by Nucleon Phytopure Plant DNA Extraction kit (Amersham Biosciences) according to the manufacturers' protocol.

Two Type III LOXs,* r9-LOX1* (accession number: AB099850) and* L-2 *(accession number: X64396), and a leaf-specific* LOX RCI-1* (accession number: AJ270938), as well as* RCI-1* (accession number: AJ270938) were selected for study. Interestingly, the L-3 (accession number: E03480) sequence was noted to be identical to the L-2 sequence (accession number: X64396) known to be distributed in rice embryo and have C9 specific LOX activity [13]. The nucleotide sequences of* L-2* and* L-3* (Type III) were identical. The primers for these* LOX* genes were listed in Table 1. Gene sequences for inverted repeats were amplified using thermostable, proofreading DNA polymerase from Stratagene (La Jolla, CA, USA). The CACC sequence was added to the 5′ end of forward primer for facilitating directional incorporation into Invitrogen's pENTR/D-TOPO entry vector. PCR was performed by using KOD-Plus-Neo (TOYOBO, Japan) and then the products were separated by 0.7% agarose gel. The target fragments were collected using Qiangen gel purification kit.

### 2.3. Construction of hpRNAi Vectors

RNAi constructs were prepared following the Gateway System protocol [21]. For constructing hpRNAi vectors, the target gene fragments were firstly cloned into TOPO pENTR following the Gateway System protocol (Invitrogen, Carlsbad, CA). The transformation of* Agrobacterium tumefaciens* EHA105 with the pANDA vector was performed using the Freeze and Thaw method [22].* Agrobacterium*-mediated transformation in rice was carried out as described previously [23, 24], with minor modifications [25]. The positive control EHA105 was transformed with pWHNG vector.

### 2.4. Rice Transformation

The transformation of* Agrobacterium tumefaciens* EHA105 with the pANDA vector was performed using the Freeze and Thaw method [22].* Agrobacterium*-mediated transformation in rice was carried out as described previously [23, 24], with minor modifications [25]. The positive control EHA105 was transformed with pWHNG vector.

Transformation was carried out using 21-day-old calli grown on B5 medium with 2.2 mg/L 2, 4 D. After being treated with liquid cocultivation medium (YEP containing EHA105 at 600OD of 1) for 20 min, the calli were transferred to a solid cocultivation medium (B5 medium with 2.2 mg/L 2, 4 D and 100 um acetosyringone) for 3 days. After cocultivation, the transformed calli were cultured in solid B5 medium with 2.2 mg/L 2, 4 D and 250 mg/L Claforan for 7 days and then transferred to the selective medium (B5 medium with 2.2 mg/L 2, 4 D, 250 mg/L Claforan, and 25 mg/L geneticin) for 15 days. The proliferated, yellowish white calli were transferred to the same medium for another 15 days. Resistant calli were transferred to regeneration medium (B5 medium with 0.03 mg/L Picloram and 0.35 mg/L BA) for 15 days. After 15 days, the medium was replaced with fresh medium for another 15 days. Seedlings were then transferred to fresh B5 medium without antibiotics for 15 days and then transferred to the planters in greenhouse.

### 2.5. Real-Time PCR

Total RNA was extracted from mature seed, 2-day-old germinating seed, imbibed seed, and stabilized bran and 35-day-old regeneration plants of different varieties rice or transgenic plants using Trizol (Invitrogen, Carlsbad, CA) [26] and treated with RNase-free DNase I (TaKaRa, Shuzo, Kyoto, Japan) to remove potential contaminating genomic DNA. First Strand cDNA was synthesized using SuperScript TM II RT kit (Invitrogen, Carlsbad, CA).

All samples were amplified in triplicate from the same RNA preparation and the mean values were calculated. The primers for real-time analysis were designed based on gene specific sequences (see Supplementary Table S1 in Supplementary Material available online at http://dx.doi.org/10.1155/2016/4275904). RT-PCR was performed by using Syber Green® (Roche Diagnostics) on C1000*™* Thermal Cycler equipped with CFX96*™* Real-Time System (Bio-Rad, USA). Primer specificity was tested by isolated PCR products in high resolution gel electrophoresis. Melting curve program (60–95°C) was performed with the heating rate at 0.10°C/sec, continuous fluorescence measurement, and a final cooling step to 40°C.

To determine relative amount of* LOXs* mRNA transcript in different rice tissues, we applied the REST (Relative Expression Software Tool) calculations based on the Ct (cycle threshold) values at a constant fluorescence level [27, 28]. The relative expression/mRNA transcript ratio of each* LOX* was computed based on its RT-PCR efficiency and the crossing point difference between* LOX* and* Actin *control.

### 2.6. SPME GC/MS Analysis

Samples for SPME GC/MS were prepared by first hulling the seeds using hands or table top rice huller depending on the amount of rice and the easiness to hull the seeds. The hulled seeds were then milled to 10% (removal of bran layer) degree using “WonderHand Mill” [29]. The native and transgenic bran was subjected to identical storage conditions to maintain identical moisture level. SPME GC/MS analysis was performed using a method modified by Grimm et al. [30].

## 3. Results

### 3.1. RNAi Vector Construct and Development of Transgenic Plants

**Figure 1:**
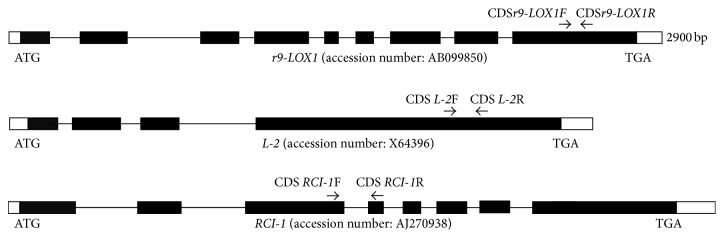
Gene structure and the target site for RNA interference.

**Figure 2:**
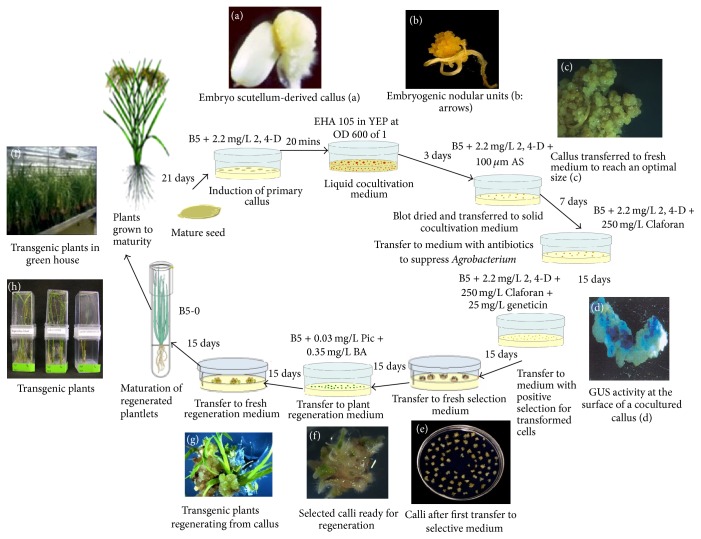
*Agrobacterium*-mediated transformation. The figure is a schematic diagram of the events involved in rice transformation.

PCR results showed the correct sizes of* LOX* fragments with the range of 200–300 bp for* 9r-LOX1 *and* L-2* (Supplementary Figure S1). The fragments of LOX genes were targets for RNAi shown as Figure 1. These fragments were initially linked to TOPO pENTR vector and then transferred into pANDA for hpRNAi expression (Supplementary Figure S2). The insertions of target fragments in pENTER vector and in pANDA were confirmed by PCR and restriction enzyme digestion (Supplementary Figures S3 and S4). Sequencing results revealed that all the sequences for* r9-LOX1*,* L-2*, and* RCI-1* were consistent with the original sequences in the database only with a few mismatches. Moreover, the binary pWHNG vector containing* GUS* was chosen as positive control of* Agrobacterium*-mediated transformation (Supplementary Figure S5). The hpRNAi vector and pWHNG were transformed into rice by* Agrobacterium*-mediated transformation of rice mature embryo. A total of 160 to 200 explants were used for* r9-LOX1*,* LOX2*, or* RCI-1 *gene silencing plants and 40 explants were for generation of “positive control” transgenic plants. After selection and regeneration, 12 to 22 transformed T_0_ plants were produced for hpRNAi vectors and four for pWHNG vector, with average regeneration rate of 8.78%. The transgenic plants were further confirmed by PCR using vector specific primers (Supplementary Figure 6 and Table 2). The result showed that two to seven PCR T_0_ plants were positive for the vectors, with average rate of positive confirmation of 3.25%. The plants were grown in greenhouse until mature, and then T_1_ seeds were harvested and grown for two generations to obtain stable transgenic plants. The different tissues were collected for the expression analysis of target gene. Schematic diagram for the transformation process is depicted in Figure 2.

### 3.2. Expression Pattern of* LOXs*

**Figure 3:**
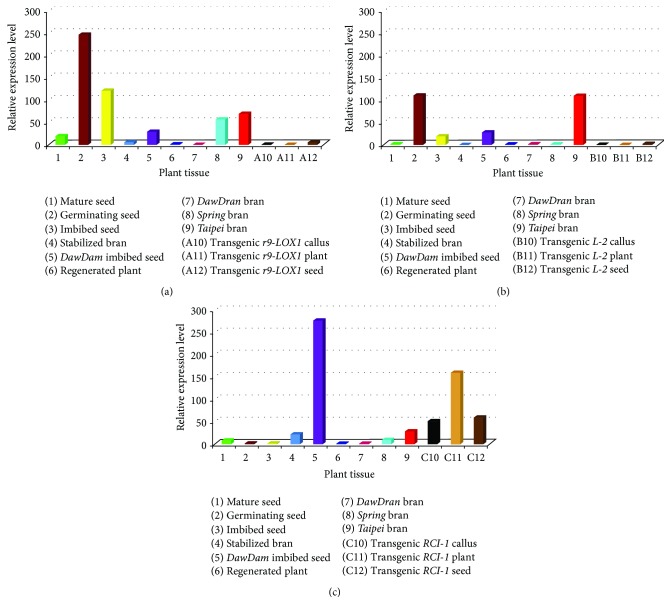
Relative expressions of three* LOX* genes (a)* r9-LOX1*, (b)* L-2*, and (c)* RCI-1*. among transgenic and nontransgenic rice tissues.

This study analyzed the expression patterns of two bran/seed-specific genes,* r9-LOX1* and* L-2*, and one chloroplast-specific gene,* RCI-1* (Figures 3(a), 3(b), and 3(c)).* r9-LOX1 *was highly expressed in germinating and imbibed seed, as well as* Spring* and* Taipei* bran.* L-2* was also highly expressed both in germinating and imbibed seed, as well as* Taipei* bran. In contrast,* r9-LOX1* and* L-2* were both barely expressed in transgenic tissues.* RCI-1* was highly expressed in “null” mutant “*DawDam*” imbibed seed, as well as transgenic tissue.

The expressions of these three* LOXs* showed tissue/cultivar-specificity on a certain level (Figures 3(a), 3(b), and 3(c)).* r9-LOX1* expression was generally much higher in active bran/seed than in stabilized bran, mature seed, and regenerated plant. Among active bran/seed tissue, germinating seeds had the highest expression of* r9-LOX1*, followed by imbibed seed,* Taipei* bran, and* Spring* bran. Similarly,* L-2* had relative high expression in active bran/seed tissue. Interestingly, the chloroplast-specific* RCI-1* showed exceedingly high expression in the imbibed seeds of the null mutants “*DawDam*” and in all of the transgenic calluses, plants, and seeds (Figure 3(c)).

### 3.3. The Volatile Byproducts in* r9-LOX1* RNAi Rice Bran

**Figure 4:**
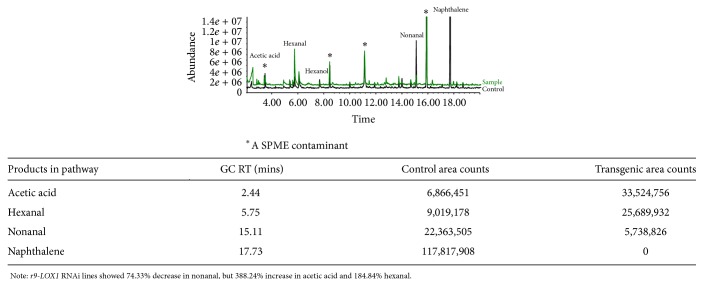
SPME/GC-MS analysis of the volatile byproducts by lipid oxidation in nontransgenic and transgenic rice bran of* r9-LOX1*.

The volatile byproducts during lipid oxidation in nontransgenic and transgenic RNAi rice bran were examined by SPME/GC-MS. Acetic acid and hexanal are the direct products of 13-lipoxygenase, while nonanal is formed during the oxidation of linoleic acid by LOX. In SPME/GC-MS analysis, silencing* r9-LOX1* coding region was obviously associated with the changes of byproducts during lipid oxidation. The contents of acetic acid were much higher in transgenic bran than in the control lines by 388.24%, as well as hexanal by 184.84%. In contrast, the contents of nonanal were significantly decreased in transgenic lines by 74.33% (Figure 4).

## 4. Discussion

### 4.1. RNAi Approach for Nutrition Improvement and Genetic Study in Crops

HGS-hpRNAi has been used to improve nutritional qualities in several crops. For example, it is used to successfully reduce *γ*-Gliadins in bread wheat [31]. Besides, it is used to reduce the expression levels of two starch-branching enzymes, SBEIIa and SBEIIb, which results in increased amylose content [32]. In transgenic peanut, knocking down* oleate desaturase* (*FAD2*) by RNAi can increase oleic acid content by 70% [33]. Moreover, the application of this technology in our research could provide more insights into the functional characterization of rancidity-associated* LOXs*. Traditionally, studies of gene functions mainly relied on traditional breeding strategies which are time consuming. In the recent decade, RNAi have largely expedited this process. In this study, pANDA vector was adopted, since it is an efficient RNAi vector for rice and has been used for* Agrobacterium* transformation in rice over the years [21]. Our results showed that two bran/seed-specific LOXs' expression was suppressed in more than 90% of the transgenic plants, indicating the high efficiency of RNAi system.

### 4.2. LOXs' Expression Patterns

The expression of two bran/seed-specific* LOXs* and one plastid-specific* LOX* were studied among different tissues. Two bran/seed-specific genes,* r9-LOX1* and* L-2*, were both highly expressed in active seed including imbibed and germinating seed but barely expressed in stabilized bran, mature seed. In previous studies, it is reported that* r9-LOX1* is expressed in rice imbibed seeds [13] and in tea plant seeds [34]. The similar expression pattern of the two bran/seed-specific LOXs may be due to their similar biological functions in rice bran/seeds and 71.3% homology [13]. Compared to* r9-LOX1*,* L-2* showed about 15% decreased expression in germinating and imbibed seeds and less expression in* Spring* bran. The results are consistent with the previous study where an expressional variation is observed between the predicted bran/seed-specific* LOXs* in soybean [35]. The different expression patterns of LOX isozymes indicate that they may also play other different roles during seed developmental stages.

The expression patterns of* r9-LOX1* and* L-2* were significantly different from that of* RCI-1* (Figures 3(a), 3(b), and 3(c)). The expressions of the two bran/seed-specific* LOXs* were low in null-mutation imbibed seed and rare in the transgenic T_1_ seeds with corresponding RNAi construct (Figures 3(a), 3(b), and 3(c)). Interestingly,* RCI-1* is previously reported to be chloroplast-specific [36]. The chloroplast-specific* RCI-1 *showed an exceedingly high expression in the imbibed seeds of the null mutants as well as an expression at a certain level in* Taipei* bran (Figure 3(c)), which indicates that we should take* RCI-1* as plastid-specific rather than the previously reported leaf-specific. In addition,* RCI-1* expression was also observed in the seeds of transgenic lines (Figure 3(c)). Compared to* r9-LOX1* and* L-2*,* RCI-1* showed a high expression level in the imbibed seeds of null-mutation line* DawDam*, which might be caused by a mechanism of negative feedback between Type III LOX members in seeds.

### 4.3. The Biologic Functions of* r9-LOX1*

In SPME/GC-MS analysis,* r9-LOX1* RNAi line showed 74.33% reduction of nonanal (formed during the oxidation of linoleic acid by LOX). These results indicate that* r9-LOX1* positively regulates the amount of nonanal. Previous study also discovers a positive correlation between nonanal concentration and LOX activity [37]. However,* r9-LOX1* RNAi lines showed 388.24% increase in the amount of acetic acid and 184.84% increase in hexanal (direct products of 13-LOX) (Figure 3), which may be due to a negative feedback mechanism between* LOX *family members. Similarly,* RCI-1* RNAi line did not exhibit efficient silencing effect, which may be due to the shared oxylipin synthesis domain between Type III and Type II LOXs [38]. These results suggest that the LOXs derived from different categories may coordinate with each other and play important roles in many biological processes. For example, the complete knockdown of Type III* LOXs* results in poor defense function and therefore leads to poor agronomic traits in* DawDam*.

## 5. Conclusion

The results suggest that* r9-LOX1* plays roles in synthesis pathways of nonanal, acetic acid, and hexanal in rice bran/seed. Notably, it is very hard to reduce all of* LOX* rancid byproducts by silencing* LOXs*, because of the complementary action of different members of* LOX* family. Since a number of* LOXs* also play crucial parts in some basic biological processes, silencing all of them in rice will result in plants with poor agronomic characters, like* DawDam*. The information will help understand the function of* LOXs* and improve the storage quality in the future.

## Supplementary Material

Figure S1: Amplification of lipoxygenase fragments in rice.
Figure S2: Structure of the pANDA vector and procedure for RNAi vector construction. (A) The sequence of a gene used for inverted repeats (IR) is amplified by PCR using primers. The CACC sequence is added to 5′ end of forward primer for providing the correct direction to the PCR product. (B) The overhang in the the pENTR/D-TOPO cloning vector (GTGG) invades the 5′ end of the PCR product, anneals to the added bases, and stabilizes the PCR product in the correct orientation. The entry clone containing the PCR product is completed. (C) The final RNAi vector was produced by an LR clonase reaction between the entry clone and pANDA. PANDA vectors contain the promoter and IR regions. The LR clonase reaction site *att*R is located at both sides of the *gus* linker in the antisense and sense orientations. The pANDA vector is the binary vector for Agrobacterium mediated transformation and has kanamycin and hygromycin resistance marker genes.
Figure S3: Transformation into pENTR/D TOPO and verification of inserted DNA fragments by PCR and restriction enzymes. The transformation is checked using gene specific primers for (A) CDS-*r9-LOX1*, CDS-*L-2*, and CDS-*RCI-1*. The enzymes digested the inserted DNA (B) CDS-*r9-LOX1*, (C) CDS-*L-2*, (D) CDS-*RCI-1*. In this case NotI and AscI are the special sites.
Figure S4: Transformation of *r9-LOX1* insert in pANDA: Complete transformation of *r9-LOX1* insert in pANDA vector was verified by PCR and restriction digestion using Kpn1 and Sac1.
Figure S5: (A) Regeneration control; (B) GUS positive control (*N. xanthae*); (C) construct EHA105+pWHNG; (D) EHA105+pANDA *r9-LOX1*; (E) GUS possitive control (*N. xanthae*) roots.
Figure S6: Transformation of insert *r9-LOX1* in Agrobacterium (EHA105 strain). (A) PCR was used to confirm *r9-LOX1*, Gus linker (GL) and neomycin phosphotransferase (NPTII) in pANDA (B) PCR was used Transformation of rice using pWNHG vector, Rice (c.v. *Taipei-309* and c.v. LaGrue) was transformed with a construct (pWNHG) that carried genes coding for nptII, hygromycin phosphotransferase (Hyg), and p-glucuronidase (GUS). This served as the positive control (C) The regenerated plant.
Table S1: Primer sequences of q-PCR for three LOX genes.

## Figures and Tables

**Table 1:** Primer sequences of three LOX genes for cloning.

Gene (accession number)	Primers	Sequence	Product size
*r9-LOX* (AB099850)	CDS *r9-LOX1*F	CACCGCTGGACGAGAACCCAGAGAAG	238 bp
	CDS *r9-LOX* 1R	CAGGGGGTCCTTGTTCATGTT	
*RCI-1* (AJ270938)	CDS*RCI-1*F	CACCCGAAGGCTACTTCAGGGAGGTG	214 bp
	CDS*RCI-1*R	CGTGTCCCTGACAAGTTGGATG	
*L-2* (X64396)	CDS*L-2*F	CACCCCTCGCCATCTACTACCCCAACG	217 bp
	CDS*L-2*R	GTACGGGTACTGCCCGAAGTTG	

**Table 2:** Rate of *Agrobacterium*-mediated transformation.

Constructs	Total explants	Transformed plants	Regeneration percentage (%)	Confirmed transformation	Transformed plants percentage (%)
Negative control	7	4	57.0		
EHA105 + pWHNG	40	4	10.0	2	5.0
EHA105 + *r9-LOX*	160	12	7.5	4	2.0
EHA105 + *L2*	200	22	11.0	7	3.0
EHA105 + *RCI-1*	180	12	6.6	5	3.0
Mean			8.78%		3.25%
