# Supplementary material for: Functional Characterization of 9-/13-LOXs in Rice and Silencing Their Expressions to Improve Grain Qualities
Source: Biomed Res Int. 2016 Jun 15;2016:4275904. doi: 10.1155/2016/4275904 (PMC4925972; doi:10.1155/2016/4275904)
Supplement: Supplementary file 1 — Figure S1: Amplification of lipoxygenase fragments in rice. Figure S2: Structure of the pANDA vector and procedure for RNAi vector construction. (A) The sequence of a gene used for inverted repeats (IR) is amplified by PCR using primers. The CACC sequence is added to 5′ end of forward primer for providing the correct direction to the PCR product. (B) The overhang in the the pENTR/D-TOPO cloning vector (GTGG) invades the 5′ end of the PCR product, anneals to the added bases, and stabilizes the PCR product in the correct orientation. The entry clone containing the PCR product is completed. (C) The final RNAi vector was produced by an LR clonase reaction between the entry clone and pANDA. PANDA vectors contain the promoter and IR regions. The LR clonase reaction site attR is located at both sides of the gus linker in the antisense and sense orientations. The pANDA vector is the binary vector for Agrobacterium mediated transformation and has kanamycin and hygromycin resistance marker genes. Figure S3: Transformation into pENTR/D TOPO and verification of inserted DNA fragments by PCR and restriction enzymes. The transformation is checked using gene specific primers for (A) CDS-r9-LOX1, CDS-L-2, and CDS-RCI-1. The enzymes digested the inserted DNA (B) CDS-r9-LOX1, (C) CDS-L-2, (D) CDS-RCI-1. In this case NotI and AscI are the special sites. Figure S4: Transformation of r9-LOX1 insert in pANDA: Complete transformation of r9-LOX1 insert in pANDA vector was verified by PCR and restriction digestion using Kpn1 and Sac1. Figure S5: (A) Regeneration control; (B) GUS positive control (N. xanthae); (C) construct EHA105+pWHNG; (D) EHA105+pANDA r9-LOX1; (E) GUS possitive control (N. xanthae) roots. Figure S6: Transformation of insert r9-LOX1 in Agrobacterium (EHA105 strain). (A) PCR was used to confirm r9-LOX1, Gus linker (GL) and neomycin phosphotransferase (NPTII) in pANDA (B) PCR was used Transformation of rice using pWNHG vector, Rice (c.v. Taipei-309 and c.v. LaGrue) was trans [file 4275904.f1.zip › FigS2.doc]

**Supplementary Fig. S2 Structure of the pANDA vector and procedure for RNAi vector construction. (A)** The sequence of a gene used for inverted repeats (IR) is amplified by PCR using primers. The CACC sequence is added to 5’ end of forward primer for providing the correct direction to the PCR product. **(B)** The overhang in the the pENTR/D-TOPO cloning vector (GTGG) invades the 5’end of the PCR product, anneals to the added bases, and stabilizes the PCR product in the correct orientation. The entry clone containing the PCR product is completed. **(C)** The final RNAi vector was produced by an LR clonase reaction between the entry clone and pANDA. PANDA vectors contain the promoter and IR regions. The LR clonase reaction site *att*R is located at both sides of the *gus* linker in the antisense and sense orientations. The pANDA vector is the binary vector for *Agrobacterium* mediated transformation and has kanamycin and hygromycin resistance marker genes.

attL1

GTGG

attL2

TOPO

TOPO

pUBQ

ccd B

attR2

attR1

ccd B

attR1

attR2

T

pANDA

GUS linker

ccd B

attP1

attP2

attL1

GTGG

CACC

attL2

gene

gene

attL1

attL2

Entry clone

C. Cloning into pANDA vector

A. PCR

B. Sub-cloning into entry vector

gene

CACC

pENTR

KpnI

LR clonase mix

SacI

KpnI

SacI

pUBQ

gene

attB2

attB1

gene

attB1

attB2

T

GUS linker

1 Kb
